# Ambient Particulate Matter Exposure Impairs Gut Barrier Integrity and Disrupts Goblet Cell Function

**DOI:** 10.3390/biomedicines13081825

**Published:** 2025-07-25

**Authors:** Wanhao Gao, Wang Lin, Miao Tian, Shilang Fan, Sabrina Edwards, Joanne Tran, Yuanjing Li, Xiaoquan Rao

**Affiliations:** 1 Division of Cardiology, Department of Internal Medicine, Tongji Hospital, Tongji Medical College, Huazhong University of Science and Technology, 1195 Jiefang Ave, Wuhan 430030, China; gaohaoemail@163.com (W.G.);; 2 Oregon Institute of Occupational Health Sciences, Oregon Health & Science University, Portland, OR 97239, USA; 3 College of Osteopathic Medicine, Pacific Northwest University of Health Sciences, Yakima, WA 98901, USA; 4 Division of Cardiology, The First Affiliated Hospital of Chongqing Medical University, Chongqing 400016, China

**Keywords:** air pollution, particulate matter, colitis, inflammatory bowel disease, colon cancer, mucus barrier, goblet cell

## Abstract

**Background**: As a well-known environmental hazard, ambient fine particulate matter (PM_2.5_, aerodynamic diameter ≤ 2.5 µm) has been positively correlated with an increased risk of digestive system diseases, including appendicitis, inflammatory bowel disease, and gastrointestinal cancer. Additionally, PM_2.5_ exposure has been shown to alter microbiota composition and diversity in human and animal models. However, its impact on goblet cells and gut mucus barrier integrity remains unclear. **Methods**: To address this, 8-week-old male and female interleukin-10 knockout (IL10^−/−^) mice, serving as a spontaneous colitis model, were exposed to concentrated ambient PM_2.5_ or filtered air (FA) in a whole-body exposure system for 17 weeks. Colon tissues from the PM_2.5_-exposed mice and LS174T goblet cells were analyzed using H&E staining, transmission electron microscopy (TEM), and transcriptomic profiling. **Results**: The average PM_2.5_ concentration in the exposure chamber was 100.20 ± 13.79 µg/m^3^. PM_2.5_ exposure in the IL10^−/−^ mice led to pronounced colon shortening, increased inflammatory infiltration, ragged villi brush borders, dense goblet cells with sparse enterocytes, and lipid droplet accumulation in mitochondria. Similar ultrastructure changes were exhibited in the LS174T goblet cells after PM_2.5_ exposure. Transcriptomic analysis revealed a predominantly upregulated gene expression spectrum, indicating an overall enhancement rather than suppression of metabolic activity after PM_2.5_ exposure. Integrated enrichment analyses, including GO, KEGG, and GSEA, showed enrichment in pathways related to oxidative stress, xenobiotic (exogenous compound) metabolism, and energy metabolism. METAFlux, a metabolic activity analysis, further substantiated that PM_2.5_ exposure induces a shift in cellular energy metabolism preference and disrupts redox homeostasis. **Conclusions**: The findings of exacerbated gut barrier impairment and goblet cell dysfunction following PM_2.5_ exposure provide new evidence of environmental factors contributing to colitis, highlighting new perspectives on its role in the pathogenesis of colitis.

## 1. Introduction

Air pollutants such as nitrogen oxides, carbon monoxide, sulfur dioxide, ozone, and particulate matter (PM) [1] have emerged as significant health hazards [2,3,4] and are attributed to increased vehicle emissions and fuel consumption globally. Among these pollutants, ambient fine particulate matter (aerodynamic diameter ≤ 2.5 µm, PM_2.5_) poses the greatest health risk [5,6]. PM_2.5_ consists of diverse components, including organic chemicals, nitrate, sulfate, metals, soot, and crustal elements, originating from various sources, such as industrial emissions, vehicle exhaust, windblown soil, and road dust [7,8,9].

In 2019, the global average urban PM_2.5_-attributable mortality rate was 45 to 77 (95% CI) premature deaths per 100,000 inhabitants [10]. Ischemic heart disease, stroke, and pulmonary disease have been identified as the leading mortality causes attributable to PM_2.5_ exposure in epidemiological studies [11]. A 10 µg/m^3^ rise in PM_2.5_ levels has been linked to a 0.29% rise in overall non-accidental mortality and a 0.22% increase in respiratory disease-related deaths [12]. Animal studies have demonstrated that PM_2.5_ is deposited in the terminal bronchioles and alveoli upon inhalation, triggering inflammation through the recruitment of eosinophils, neutrophils, macrophages, and T cells, ultimately leading to the destruction of the alveolar wall and lung dysfunction [13,14]. Furthermore, PM_2.5_ may act as an environmental antigen and trigger inflammatory responses in the immune system through the TLR2/TLR4/MyD88 signaling pathway, thereby exacerbating allergic pulmonary inflammation [15].

While the respiratory and cardiovascular effects of PM_2.5_ are well established, its broader systemic impacts need further investigation, as it also affects other distant organs and systems, including the gastrointestinal system, brain, and reproductive system [16]. Fine PM_2.5_ particles can penetrate the lungs and reach remote organs via the bloodstream [17]. Additionally, PM_2.5_ particles can heavily contaminate food and water and enter the human digestive system orally. In addition, these particles are cleared from the lungs by the mucociliary escalator after inhalation and are subsequently swallowed. This means that a significant portion of inhaled pollutants ultimately reaches the intestinal tract [18,19,20]. Recent research indicates that exposure to PM_2.5_, whether short-term or long-term, is associated with a higher risk of developing inflammatory bowel disease (IBD) [21,22,23]. The underlying mechanisms are highly complex. Several interacting factors are related to this pathological process, including the microbial composition, oxidative stress, lipid metabolism, immune disorders, and intestinal permeability, all of which play significant roles [24].

A multilayer mucous barrier and well-structured epithelial cells are essential for maintaining intestinal homeostasis. The intestinal epithelial layer, which includes absorptive epithelial cells (enterocytes), tuft cells, goblet cells, Paneth cells, and M cells, serves as the primary physical barrier [25]. Among these cells, goblet cells play a vital role in enteric immune defense by secreting mucins to form the protective mucous barrier [26]. However, the relationship between PM_2.5_ exposure and the disruption of the intestinal mucus barrier remains poorly understood and requires further research.

Using a State-of-the-Art whole-body small-animal PM_2.5_ inhalation exposure system, we examined the impacts of PM_2.5_ on gut inflammation and the mucous barrier in IL-10 knockout (IL10^−/−^) mice, a widely used mouse model for spontaneous colitis. Our findings demonstrated exacerbated gut barrier dysfunction following PM_2.5_ exposure, as evidenced by increased colon shortening, enhanced inflammatory cell infiltration in the mucosa, disrupted villi brush borders, and abnormal goblet cells. Additionally, transmission electron microscopy and transcriptomic analysis revealed mitochondrial damage and the involvement of redox signaling in PM_2.5_-induced goblet cell dysfunction.

## 2. Materials and Methods

### 2.1. Animals

The Institutional Animal Care and Use Committee (IACUC) at Oregon Health and Science University (OHSU) approved all animal experiments. Sixteen male and ten female 8-week-old IL-10^−/−^ mice on a C57BL/6J background were randomly assigned to either the filtered air (FA) or particulate matter (PM_2.5_) groups. When not undergoing exposure, the mice were housed in OHSU animal facilities on a regular diet.

#### ARRIVE Statement

All animal experiments and reporting complied with the ARRIVE guidelines and followed the National Research Council’s Guide for the Care and Use of Laboratory Animals.

### 2.2. Exposure Protocol

The exposure was conducted using a State-of-the-Art whole-body exposure system (Versatile Aerosol Concentrator and Enrichment System, VACES), as previously described [27,28,29]. The animals were exposed for 6 h per day, 5 days a week, over a period of 17 weeks, consistent with our previous exposure protocol. Briefly, ambient air was diverted into two paths: one was filtered through a HEPA filter and directed into FA-exposure chambers, while PM_2.5_ particles were concentrated 8 to 10 times in the other path before being directed into the PM_2.5_-exposure chambers. Local atmospheric temperature and humidity were monitored according to The Weather Channel (https://weather.com; accessed on 1 November 2019), and PM_2.5_ concentrations were recorded as detailed previously [27,28,29,30]. The mice were weighed weekly before and during the exposure.

### 2.3. Colon Histology and Staining

At the experimental endpoint, the mice were anesthetized and euthanized, and the colon was rapidly dissected. After the removal of pericolonic fat, tissues were fixed in 4% paraformaldehyde for 24 h, followed by graded dehydration, xylene clearing, and paraffin embedding. Paraffin-embedded colon samples were sectioned into 5 µm thick slices and mounted onto charged slides, and then stained with hematoxylin and eosin (H&E) for histopathological analysis. The sections were dewaxed in xylene, rehydrated using graded ethanol, stained with hematoxylin and eosin, dehydrated again, cleared in xylene, and mounted with resin and a coverslip for microscopy. Images were captured using a Keyence microscope and analyzed with its software. For pathological assessment, five representative images per sample were randomly selected, and cellular morphology, tissue architecture, and inflammatory infiltration were evaluated.

### 2.4. Quantitative Real-Time Polymerase Chain Reaction (qRT-PCR)

Total RNA was extracted from snap-frozen intestinal tissues using a QIAGEN RNeasy Mini Kit (Qiagen, Germantown, MD, USA). cDNA was synthesized using the High-Capacity cDNA Reverse Transcription Kit (Applied Biosystems, Foster City, CA, USA). The quantity and quality were tested by spectrophotometry with the NanoDrop One (ThermoFisher, Waltham, MA, USA) for both total RNA and cDNA. Quantitative PCR was conducted on an ABI QuantStudio instrument (Applied Biosystems, Foster City, CA, USA). The primer sequences are listed in Supplementary Table S1.

### 2.5. Cell Culture of LS174T

LS174T is a goblet cell line derived from human colorectal adenocarcinoma [31]. LS174T cells were purchased from Otwo Biotech (Shenzhen, China) and cultured in high-glucose Dulbecco’s Modified Eagle’s Medium (DMEM, GIBCO BRL, Gaithersburg, MD, USA), with the supplementation of 10% fetal bovine serum (FBS, Yeasen, Wuhan, Hubei, China) at 37 °C in a CO_2_ incubator. The PM standard reference material SRM 2786 was purchased from Merck Sigma (St. Louis, MO, USA). Its components have been previously reported in studies [32]. For in vitro exposure, cells in 12-well plates were treated with a complete medium containing either PBS (control group) or 100 µg/mL PM (exposure group) for 24 h. Following treatment, cells were prepared for subsequent imaging or sequencing experiments as appropriate.

### 2.6. TEM Imaging

Colon samples were processed using the Multiscale Microscopy Core at OHSU. Images were captured using an FEI Tecnai™ with iCorr™ (Integrated Light and Transmission Electron Microscope, FEI Company, Hillsboro, OR, USA).

After incubation with PM or PBS for 24 h, cultured cells were washed with PBS, followed by fixation in 2.5% glutaraldehyde for 2 h, and then in 1.0% osmium tetroxide for an additional 2 h. The samples were dehydrated through different concentrations of ethanol before being embedded in epoxy resin. After that, the samples were sectioned to a thickness of 0.5–1 µm and treated with 2% uranyl acetate and lead citrate for staining. Subsequently, a JEM-1400 transmission electron microscope was used for imaging and analysis (Jeol Ltd., Akishima, Tokyo, Japan).

### 2.7. RNA Sequencing and Analysis

The total RNA of LS174T cells was extracted using TRIzol (Invitrogen, Carlsbad, CA, USA) for RNA sequencing. It was then used as input material for cDNA library construction, and the quality of the library was further assessed by Bioanalyzer (Agilent Technologies, Santa Clara, CA, USA). An Illumina HiSeq 4000 instrument was used for sequencing by Novogene (Beijing, China). The cBot Cluster Generation System, along with the TruSeq PE Cluster Kit (Illumina, San Diego, CA, USA), was used for clustering samples as instructed by the manufacturer. Following this, an analysis of the transcriptome sequencing libraries was conducted using R (version 4.5.0, R Core Team, Vienna, Austria), and a metabolic flux analysis was performed using the R package METAFlux (version 0.0.0.9000), a transcriptomics-based flux estimation tool [33].

### 2.8. Statistical Analysis

Data were presented as mean ± standard error of the mean (SEM), unless otherwise specified. *T*-tests were used for the comparison of the means between the FA and PM groups. The qRT-PCR results were analyzed using the 2^−ΔΔCt^ method. All statistical analyses were conducted by GraphPad Prism 10.0 software (GraphPad Software, Boston, MA, USA). Statistical significance was considered when the *p*-value was less than 0.05.

## 3. Results

### 3.1. Analysis of Ambient Temperature and PM_2.5_ Concentrations in Both Ambient Air and Exposure Chamber

**Figure 1:**
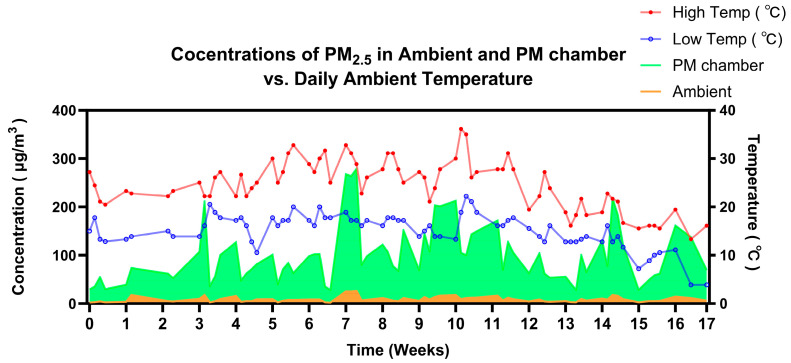
Daily ambient temperature and PM_2.5_ concentrations (µg/m^3^) in the ambient air and PM_2.5_-exposure chamber.

We employed a whole-body small-animal inhalational exposure system for in vivo PM_2.5_ exposure experiments. Local daily temperatures were obtained from The Weather Channel (https://weather.com). The temperature, ambient PM_2.5_ concentration, and concentrated PM_2.5_ levels in the exposure chamber during the 17-week exposure period are shown in Figure 1. The result indicates a positive correlation between average ambient concentrations of PM_2.5_ and the increased PM_2.5_ levels observed in the exposure chambers. Additionally, airborne PM_2.5_ concentrations were correlated with temperature, showing a positive relationship between daily high temperatures and average PM_2.5_ concentrations. The ambient average PM_2.5_ levels fluctuated within the range of 8.94 ± 1.28 µg/m^3^ (Figure 1). While the weekly average PM_2.5_ levels in the FA chambers remained below 0.5 µg/m^3^, the average PM_2.5_ levels in the PM_2.5_ chambers ranged in the region of 100.20 ± 13.79 µg/m^3^ (Figure 1).

### 3.2. Effect of Chronic PM_2.5_ Exposure on Colitis in IL10^−/−^ Mice

**Figure 2:**
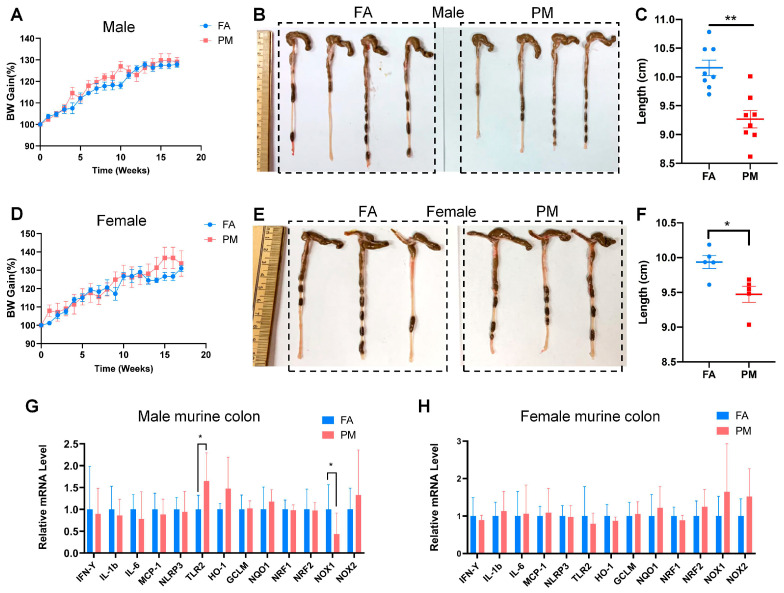
Effect of chronic PM_2.5_ exposure on IL10^−/−^ mice. (**A**,**D**) The mean body weight gains of male (**A**) and female (**D**) IL10^−/−^ mice during the 17-week exposure study. (**B**,**C**,**E**,**F**) The colon length of FA- or PM_2.5_-exposed male ((**B**) colon images and (**C**) statistical analysis) and female ((**E**) colon images and (**F**) statistical analysis) IL10^−/−^ mice. (**G**,**H**) mRNA expression levels of inflammation and redox reaction-associated genes in male (**G**) and female (**H**) murine colons tissue. Statistical significance is indicated by * *p* < 0.05 and ** *p* < 0.01.

To investigate the effect of PM_2.5_ exposure on inflammatory bowel disease, IL10^−/−^ mice were exposed to concentrated ambient PM_2.5_ or FA for 17 weeks. Pathological changes in the colon tissues were examined at the end of the experiment. No significant differences in mean body weight gain were observed between the FA and PM_2.5_ exposure groups during the exposure period (Figure 2A,D). However, the colon length in the PM_2.5_ group was significantly reduced compared to the FA group (Figure 2C,F).

RT-PCR quantification revealed an increase in *Tlr2* mRNA expression and a decrease in *Nox1* in the colon tissue of the PM_2.5_-exposed male mice. However, other inflammation-associated genes (*Ifng*, *Il1b*, *Ii6*, *Nlrp3*, and *Mcp1*) and oxidative stress-associated genes (*Ho1*, *Gclm*, *Nqo1*, *Nrf1*, *Nrf2*, and *Nox2*) showed no significant differences between the two groups. In contrast, all of these inflammation- and oxidative stress-associated genes showed similar expression levels in the colon tissue of female mice.

### 3.3. Chronic PM_2.5_ Exposure Damages the Intestinal Mucosal Barrier

**Figure 3:**
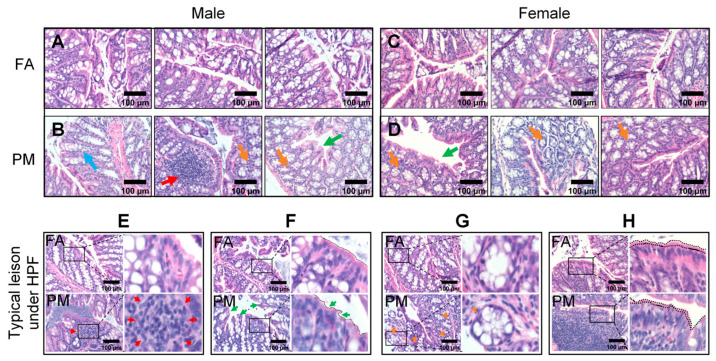
Typical histopathological alterations in PM_2.5_-exposed murine colon tissue. (**A**–**D**) H&E-stained sections from male (**A**,**B**) and female (**C**,**D**) mice. FA groups (**A**,**C**) show no obvious pathological changes. PM_2.5_-exposed groups (**B**,**D**) show varying degrees of typical damage in the intestinal epithelial tissue, including sparse enterocytes (blue arrows), increased inflammatory cell infiltration in the mucosa (red arrows), ragged brush borders of villi (green arrows), concentrated goblet cells (orange arrows), and reduced thickness of mucus layer (black dotted line). Representative images of characteristic lesions are also shown at higher magnification (**E**–**H**).

Histopathological changes in colon tissue following PM_2.5_ exposure were evaluated using H&E staining. Under the optical microscope, the colon tissue from the FA group showed an intact mucosal structure, with glandular cells arranged orderly in the crypts and fully intact villi in the colonic epithelium without significant shedding. Minimal inflammatory cell infiltration was observed in the muscular and serous layers (Figure 3A,C). In contrast, colon tissues from PM_2.5_-exposed mice exhibited pronounced histopathological alterations, including increased inflammatory cell infiltration in the mucosa (red arrows, Figure 3B,E), ragged brush borders of villi (green arrows, Figure 3B,D,F), concentrated goblet cells (orange arrows, Figure 3B,D,G), sparse enterocytes (blue arrows, Figure 3B), and a thinner mucus layer (Figure 3H). Notably, these PM_2.5_-induced histopathological changes were more severe in male mice in comparison to female mice (Figure 3).

### 3.4. Ultrastructural Characterization of Colon Epithelium in PM_2.5_-Exposed Mice

**Figure 4:**
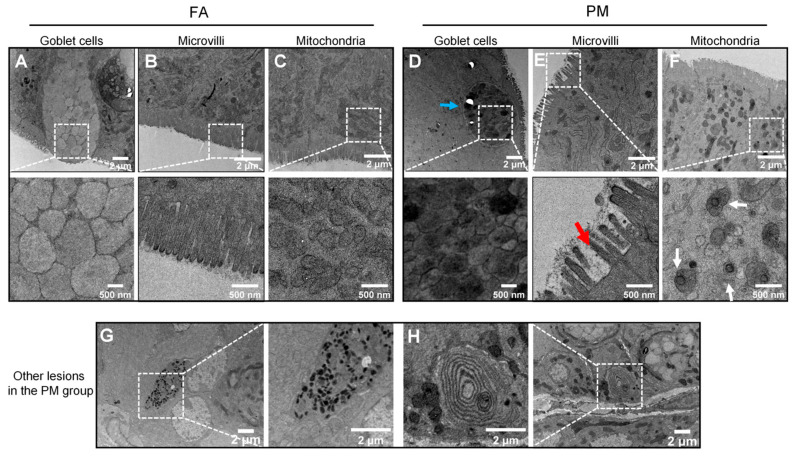
Typical ultrastructural alterations in PM_2.5_-exposed murine colon tissue. The intercellular ultrastructures of colon tissues from the FA group (**A**–**C**) and PM_2.5_-exposure group (**D**–**F**) are examined by TEM. Typical ultrastructural alterations include rounded/concentrated goblet cells (blue arrows), increased spacing and decreased length of microvilli (red arrows), and increased lipid droplets in the mitochondria (white arrows). Additionally, other abnormal pathological structures were observed exclusively in the PM group, including the deposition of electron-dense granules (**G**) and the presence of abnormal multilamellar bodies (**H**) within the intestinal epithelial tissue.

Next, we evaluated the ultrastructural alterations in the colonic epithelium of PM_2.5_-exposed mice using TEM. Compared to the controls, the most striking change in the PM_2.5_-exposed mice was that the goblet cells appeared significantly more rounded and concentrated (blue arrows, Figure 4D). Furthermore, the microvilli exhibited multiple structural abnormalities, including increased spacing and reduced length (red arrows, Figure 4E). In PM_2.5_-exposed mice, mitochondrial density was markedly reduced, while lipid droplet accumulation within the organelles was notably increased (white arrows, Figure 4F). We also found the deposition of electron-dense granules and multilamellar bodies (MLBs)—ovoid structures composed of concentric membrane lamellae—which were absent from the control samples (Figure 4G,H). These formations may be triggered by epithelial barrier disruption and reflect autophagy–lysosomal dysfunction.

### 3.5. Effect of PM_2.5_ Exposure on LS174T Goblet Cells in Vitro

**Figure 5:**
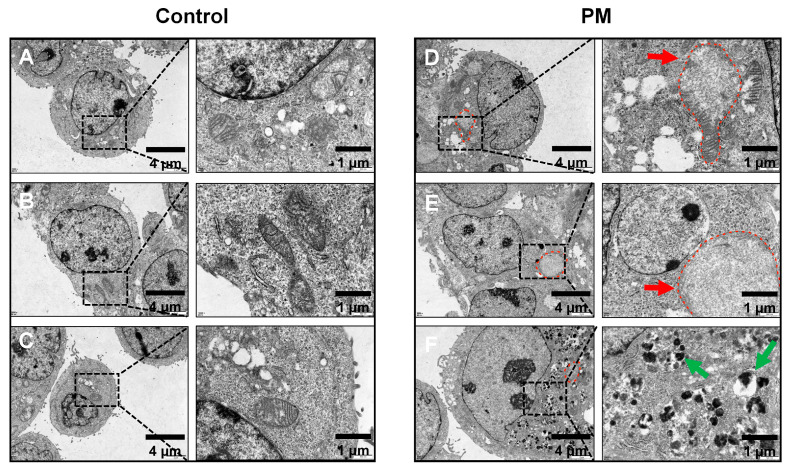
Typical ultrastructural damage in LS174T cells induced by PM exposure. Control vehicle (PBS, (**A**–**C**)) or PM-treated (**D**–**F**) LS174T cells were used for TEM examination. PM-treated cells showed swollen mitochondria (red arrows and dotted line) and an increased number of secondary lysosomes (green arrows).

LS174T is an epithelial-like cell line with goblet cell characterization. It expresses goblet cell-specific mucin precursors, including MUC2, MUC5A/C, and MUC6. Therefore, LS174T cells are widely used to study mucus activity and the regulation of mucin secretion. To examine the potential effects of PM, LS174T cells were exposed to a 100 µg/mL PM suspension in culture medium for 24 h, a concentration routinely used in in vitro PM_2.5_ exposure models. TEM was then used to observe the ultrastructural changes following the treatment. Figure 5 shows that LS174T cells without PM exposure were rounded with a few microvilli on their surface. In addition, these cells exhibited prominent mucinous secretory granules with varying electron densities (Figure 5A–C). In contrast, PM-exposed LS174T cells displayed an increase in swollen mitochondria (red arrows, Figure 5D,E) and a mass of secondary lysosomes (green arrows, Figure 5F) compared to the control. These ultrastructural changes in PM-exposed LS174T cells closely mirrored the phenotypes observed in the colons of PM_2.5_-exposed mice.

### 3.6. Gene Expression Profiles in PM_2.5_-Exposed LS174T Cells

**Figure 6:**
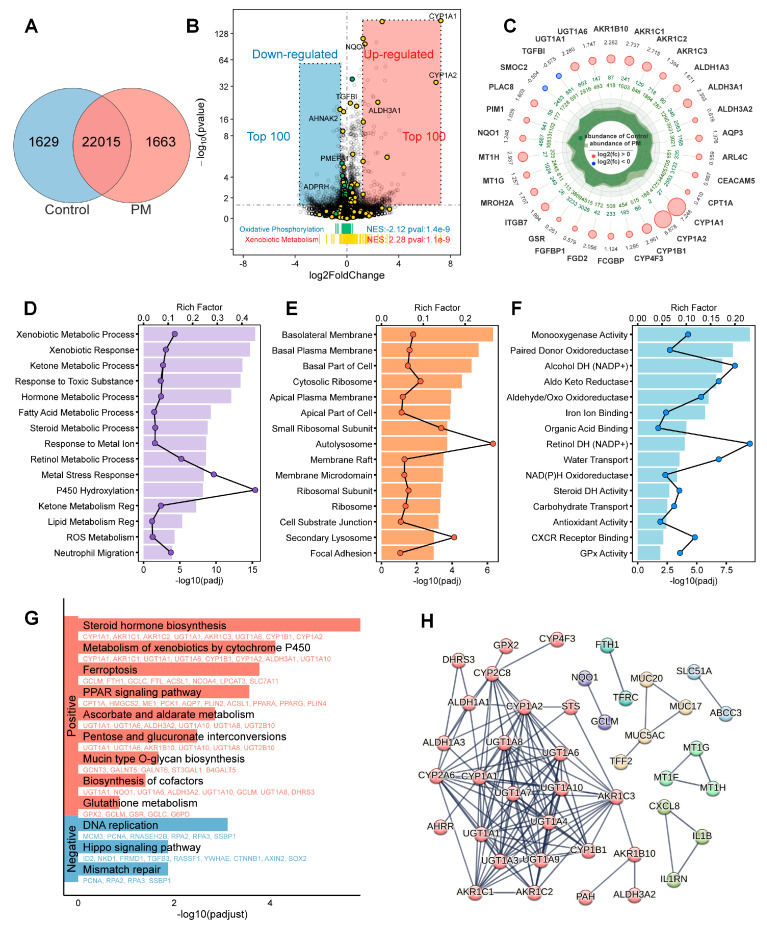
Transcriptomic analysis of LS174T cells treated with PM. (**A**) Venn diagram analysis. (**B**) Volcano plot based on differentially expressed genes. (**C**) Radar chart of top 30 differentially expressed genes by adjusted *p*-value (padj). GO enrichment analysis was performed separately for biological process (**D**), cellular component (**E**), and molecular function (**F**) categories. (**G**) KEGG pathway enrichment analysis. (**H**) Protein–protein interaction networks.

The total RNA extracted from control and PM-treated LS174T cells was subjected to RNA sequencing analysis. The correlation analysis (Supplementary Figure S1A) and PCA analysis (Supplementary Figure S1B) revealed distinct gene expression patterns between the PM and control group (Figure 6A). An analysis of differentially expressed genes (DEGs) revealed upregulated and downregulated genes in PM-exposed cells (Figure 6B). The radar chart (Figure 6C) highlights the top 30 differentially expressed genes by adjusted *p*-value (padj). The most prominently upregulated genes were significantly enriched in the xenobiotic metabolism gene set, with cytochrome P450 family proteins showing the greatest enrichment. A complete gene list is provided in the Supplementary Materials.

In the gene ontology (GO) analysis, the biological process (BP) category revealed that DEGs were predominantly enriched in pathways regulating cellular responses to exogenous stimuli or involved in metabolic synthesis and degradation. In the cellular component (CC) analysis, little overall differential enrichment was observed, with most genes localized to extracellular secretory processes. Molecular function (MF) analysis showed enrichment in pathways related to redox balance.

The KEGG pathway analysis demonstrated that the most significant pathways were upregulated, with steroid hormone metabolism emerging as the most significant, mirroring the results observed in the protein–protein interaction (PPI) analysis. Meanwhile, the DNA repair pathway was the most significantly downregulated. Taken together, these findings suggest that the heightened oxidative metabolism of xenobiotics may underlie an imbalance in oxidative stress and compromise DNA repair mechanisms.

Overall, the gene enrichment analyses suggest that the regulation of inflammation, hormone metabolism, and oxidation reduction reactions may be the main factors driving the mitochondrial dysfunction and gut barrier impairment induced by PM exposure.

### 3.7. PM Exposure Altered Metabolic Status and Promoted Oxidative Stress

**Figure 7:**
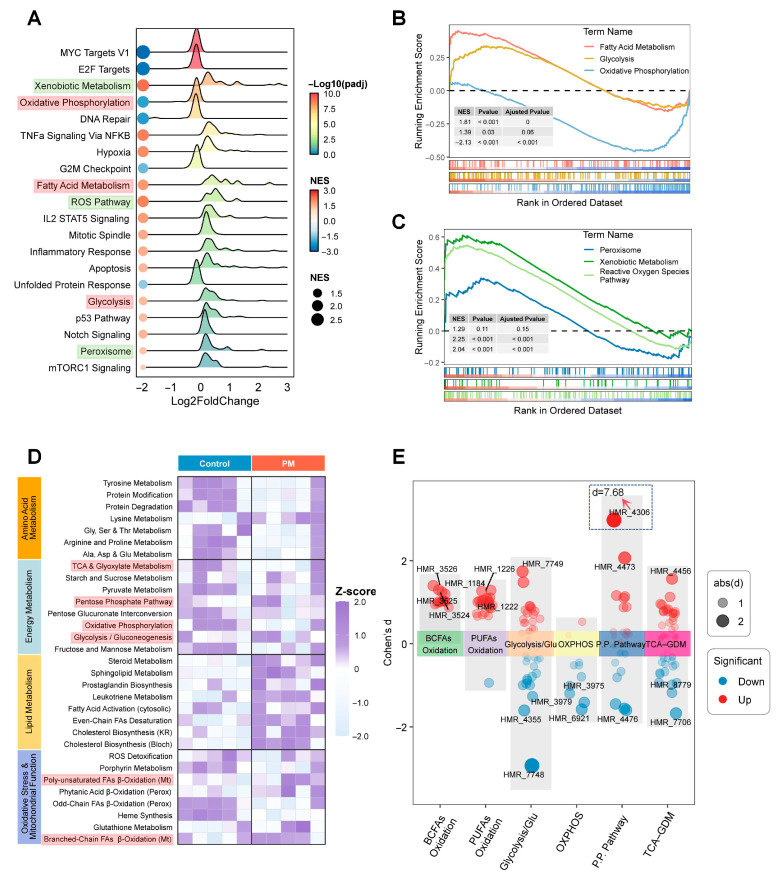
Enrichment analysis of DEGs between the PM_2.5_ treatment and control group. (**A**) Top 20 pathways enriched in the GSEA analysis (Hallmark gene sets). (**B**) GSEA highlighting differentially enriched oxidative stress-related pathways. (**C**) GSEA highlighting differentially enriched energy metabolism-related pathways. (**D**) Enrichment analysis of metabolic reaction activity predicted by METAFlux. (**E**) Changes in metabolic reaction scores related to energy metabolism and mitochondrial function (based on a genome-scale metabolic model).

Furthermore, GSEA analysis revealed that there was significant upregulation of multiple oxidative stress- and energy metabolism-related pathways (Figure 7A). Notably, pro-oxidative pathways, such as xenobiotic metabolism, peroxisome, and reactive oxygen species pathways, all showed significant enrichment (Figure 7B). In terms of energy metabolism, the most prominent features were suppressed oxidative phosphorylation and enhanced fatty acid β-oxidation in the PM group (Figure 7C).

**Figure 8:**
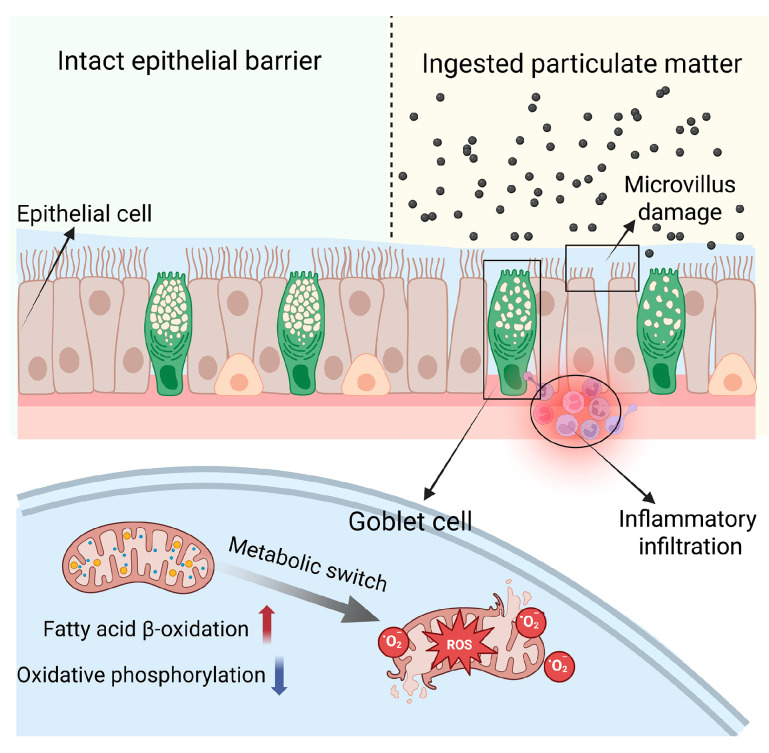
Proposed mechanism by which PM exposure induces dysfunction in goblet cells. As shown, PM inhibits mitochondrial oxidative phosphorylation (OXPHOS), leading to a compensatory increase in the β-oxidation of fatty acids. The resulting redox imbalance promotes the excessive generation of reactive oxygen species (ROS), compromising mitochondrial integrity. Cumulatively, these changes drive goblet cell dysfunction, thereby weakening the gut barrier. Created in BioRender. Sheng, L. (2025) https://BioRender.com/1zre29d.

Accordingly, we applied METAFlux—a computational framework for predicting metabolic reaction activity and fluxes—to evaluate the metabolic preference of the PM-exposed LS174T cells. After obtaining metabolite reaction activity scores from METAFlux, we performed KEGG pathway enrichment analysis based on human genome-scale metabolic model subcategories. The resulting data are displayed in Figure 7D,E. Enhanced fatty acid β-oxidation and suppressed oxidative phosphorylation were consistent with earlier analyses. Notably, the reaction HMR_4036, classified under the pentose phosphate pathway subnetwork, exhibited the most pronounced changes following PM exposure. The HMR_4306 reaction corresponds to the reversible enzymatic conversion catalyzed by glucose 6 phosphate dehydrogenase (G6PD), converting glucose 6 phosphate to 6-phosphogluconolactone and generating NADPH under normal physiological conditions. However, following PM exposure, the metabolic flux through HMR_4306 shifts from the lactone to G6P direction, consuming NADPH and thereby weakening the cellular antioxidant defense system. This renders cells more susceptible to reactive oxygen species accumulation, suggesting a potential mechanism by which PM induces oxidative damage (Figure 8).

## 4. Discussion

Air pollution, especially exposure to PM_2.5_, remains a serious public health concern worldwide [5]. The mean population-weighted ambient PM_2.5_ concentration across urban areas globally is 35 µg/m^3^ (SD 26 µg/m^3^), while PM_2.5_ levels in public spaces range from 50 to 180 µg/m^3^ [34]. In specific occupational groups, such as railway workers and bus drivers, the average daily inhaled PM_2.5_ can reach as high as 513.53 µg, which is 5–12 times higher than that of passengers [34].

PM_2.5_ is a heterogeneous mixture comprising complex components, including carbon, metals, sulfates, nitrates, and organic compounds [35,36,37]. Insoluble components of PM_2.5_, such as iron, uranium [38], and vanadium, can disturb host physiology through their redox potential and potential for direct physical injury [39,40]. Meanwhile, soluble components like lipopolysaccharides [41], polycyclic aromatic hydrocarbons (PAHs) [42], and nitro [43] act as inducers for signal transduction and inflammatory mediators, exacerbating various pathological processes. PM_2.5_ particles can not only reach the lung and remote organs via the respiratory tract and bloodstream, but they can also access the digestive tract through mucociliary clearance and oral ingestion [18,19], a pathway that is often overlooked by researchers. It has been estimated that individuals may ingest more than 10^12^ particles daily from a typical Western diet [44]. These particles can then physically adhere to epithelial cells and accumulate in macrophages of gut-associated lymphoid tissue. Additionally, particulate matter can be modified by digestive enzymes and gastric acid, altering its physicochemical properties as it travels through the gastrointestinal tract [45]. Indeed, recent studies have shown that PM_2.5_ exposure causes damage to the gastrointestinal tract [21,22,23]. Strong associations have been established between air-pollution exposure and an increased risk of conditions such as appendicitis [46], abdominal pain [47], Crohn’s disease [48], and inflammatory bowel disease [22,49]. However, the mechanisms by which PM_2.5_ induces intestinal diseases remain poorly understood.

In this study, we utilized IL-10 knockout mice, a model of spontaneous colitis, to investigate the impact of PM_2.5_ exposure on the gastrointestinal tract. This mouse strain is genetically predisposed to intestinal inflammation under normal feeding conditions [50]. The mechanisms underlying spontaneous colitis in this model include a loss of tolerance to bacterial antigens and increased intestinal permeability [51,52]. Our findings demonstrated that PM_2.5_ exposure led to a significant shortening of colon length in these animals, along with a mild upregulation of *TLR2* and *NOX1* in colon tissues. Moreover, histological examination further confirmed these effects, showing increased inflammatory cell infiltration, ragged brush borders on the villi, clustered goblet cells, and a reduced number of enterocytes in PM_2.5_-exposed mice. TEM validated these pathological alterations in the microvilli of PM_2.5_-exposed mice, including increased spacing, decreased length, and abnormal structure, which were rarely observed in the FA group. Additionally, we noted several subcellular changes in response to PM_2.5_ exposure, such as decreased mitochondria density and an increase in lipid droplets within the mitochondria. Similar changes were observed in the goblet cell line LS174T. Collectively, these results indicate that PM_2.5_ exposure exacerbates damage to the epithelial barrier in colitis.

Previous reports [53,54,55] have primarily focused on the effects of PM_2.5_ on the microbiota and intestinal epithelium, particularly concerning villous epithelial cells and intercellular junctions. However, the alterations in intestinal mucus barriers and goblet cells associated with PM exposure remain unclear. Goblet cells, which are the primary mucus-producing cells, continuously secrete mucus and are responsible for maintaining the gut mucus layer [56]. In this study, we found that the gut epithelium layer in PM_2.5_-exposed animals was thinner, despite an increased density of goblet cells. Further ultrastructural investigation revealed a decreased density of mitochondria and an accumulation of lipid droplets within the mitochondria of goblet cells. These findings suggest a dysfunction of goblet cells in response to PM_2.5_ exposure.

As mentioned earlier, PM_2.5_ particles can penetrate the intestinal mucus layer and adhere to mucosal epithelial cells. We and others have demonstrated that macrophage and lung epithelial cells can uptake PM_2.5_ particles through phagocytosis of insoluble components and/or pinocytosis of soluble components [29,30,57,58]. To investigate whether goblet cells could uptake insoluble PM_2.5_ particles, we stimulated the goblet cell LS174T with 100 µg/mL of PM particles and conducted TEM and transcriptomic analysis. Interestingly, no apparent phagocytosis of PM particles by LS174T cells was observed under TEM, likely due to the distinct cellular characteristics of goblet cells compared to macrophages. TEM analysis revealed a marked increase in secondary lysosomes in PM-exposed cells, which is an essential process for metabolizing xenobiotic toxins. Mitochondrial morphology was also notably altered, with swelling of the organelles and the loss of internal cristae.

Transcriptomic profiling after PM exposure revealed a predominantly upregulated gene expression spectrum, indicating an overall enhancement rather than suppression of metabolic activity. Integrated enrichment analyses, including GO, KEGG, and GSEA, showed that the upregulated genes were primarily enriched in pathways related to oxidative stress, xenobiotic (exogenous compound) metabolism, and lipid metabolism. In contrast, downregulated genes were significantly associated with DNA repair and oxidative phosphorylation pathways.

We employed METAFlux to predict cellular metabolic activity focused on energy supply, offering a metabolic perspective on PM’s effects. The results indicated that PM exposure suppressed oxidative phosphorylation while enhancing the β-oxidation of fatty acids, accompanied by increased NADPH consumption, inevitably exacerbating redox imbalance. These results provided further evidence supporting the findings that PM promotes the production of free radicals and induces oxidative stress in the epithelial-like goblet cells, leading to the dysfunction and increased permeability of the gut barrier [59,60].

The global incidence and prevalence of IBD have risen markedly over the past several decades, particularly in newly industrialized nations [61]. Moreover, population-based studies indicate that urban dwellers exhibit higher rates of IBD than their rural counterparts [62], underscoring the critical role of environmental factors in disease pathogenesis. Among industrial city residents chronically exposed to elevated levels of air pollution, and especially among those already afflicted with IBD, PM_2.5_ represents a significant, yet often underappreciated, risk factor for gastrointestinal morbidity [23]. In the present study, we provide compelling evidence that PM_2.5_ exposure compromises the function of the intestinal mucus barrier, driven predominantly by a complex interplay between oxidative stress and metabolic reprogramming in goblet cells. These findings have important implications for our understanding of gut health under environmental stressors. Furthermore, our work not only emphasizes the necessity of targeted clinical interventions and secondary preventive strategies, but also lays a critical foundation for future research and policy development aimed at mitigating the adverse health impacts of air pollution.

## 5. Conclusions

This study is the first to report that chronic PM exposure promotes the dysfunction of intestinal goblet cells in a mouse model of colitis, accompanied by exacerbated injury to the epithelial barrier. However, further studies are needed to determine the extent to which goblet cell dysfunction contributes to PM-associated colitis.

## Data Availability

The data are presented in the manuscript. Additional information obtained during the experiments is available upon request.

## Supplementary Materials

The following supporting information can be downloaded at https://www.mdpi.com/article/10.3390/biomedicines13081825/s1: Figure S1: Transcriptomic profiling results; Table S1: Sequences of the primers for RT-PCR.

## Abbreviations

The following abbreviations are used in this manuscript:

DEGs	differentially expressed genes
FA	filtered air
GO	gene ontology
H&E	hematoxylin and eosin
KEGG	Kyoto Encyclopedia of Genes and Genomes
MLBs	multilamellar bodies
PM	particulate matter
PPI	protein–protein interaction
qRT-PCR	quantitative real-time polymerase chain reaction
TEM	transmission electron microscopy
