# Supplementary material for: Ambient Particulate Matter Exposure Impairs Gut Barrier Integrity and Disrupts Goblet Cell Function
Source: Biomedicines. 2025 Jul 25;13(8):1825. doi: 10.3390/biomedicines13081825 (PMC12383586; doi:10.3390/biomedicines13081825)
Supplement: Supplementary file 1 [file biomedicines-13-01825-s001.zip › biomedicines-3750252-supplementary.pdf]

Supplementary Materials for

Ambient particulate matter exposure impairs gut barrier integrity and disrupts goblet cell function

Wanhao Gao *et al.*
*Corresponding author: Xiaoquan Rao, Email: xqrao@tjh.tjmu.edu.cn

This PDF file includes:

Table S1. Sequences of the primers for RT-PCR.
Figur S1. Transcriptomic Profiling Results.

Supplementary Tables and Figure

Table S1. Sequences of the primers for RT-PCR.

Primer	Forward Sequence (5′-3′)	Reverse Sequence (5′-3′)	Species
GCLM	AATCAGCCCCGATTTAGTCAG	CGATCCTACAATGAACAGTTTTGC	mouse
HO1	ACAGAGGAACACAAAGACCAG	GTGTCTGGGATGAGCTAGTG	mouse
IFNg	CCTAGCTCTGAGACAATGAACG	TTCCACATCTATGCCACTTGAG	mouse
IL-1b	ACGGACCCCAAAAGATGAAG	TTCTCCACAGCCACAATGAG	mouse
IL6	CAAAGCCAGAGTCCTTCAGAG	GTCCTTAGCCACTCCTTCTG	mouse
MCP1	GTCCCTGTCATGCTTCTGG	GCTCTCCAGCCTACTCATTG	mouse
NLRP3	CCCATGAGTTCCCTTAAGCTG	AGTGCCCAGTCCAACATAATC	mouse
Nox1	AATCCCATCCAGTCTCCAAAC	CCATAGCTGACGTTACCATGAG	mouse
Nox2	TCCTATGTTCCTGTACCTTTGTG	CCCACCTCCATCTTGAATCC	mouse
NQO1	TGAAGAAGAGAGGATGGGAGG	GATGACTCGGAAGGATACTGAAAG	mouse
Nrf1	AATGTCCGCAGTGATGTCC	GCCTGAGTTTGTGTTTGCTG	mouse
Nrf2	TCCCATTTGTAGATGACCATGAG	CCATGTCCTGCTCTATGCTG	mouse
TLR2	GGCCATAACAGTCCTCTTCAG	GACAGGTCAAGGCTTTTCATG	mouse

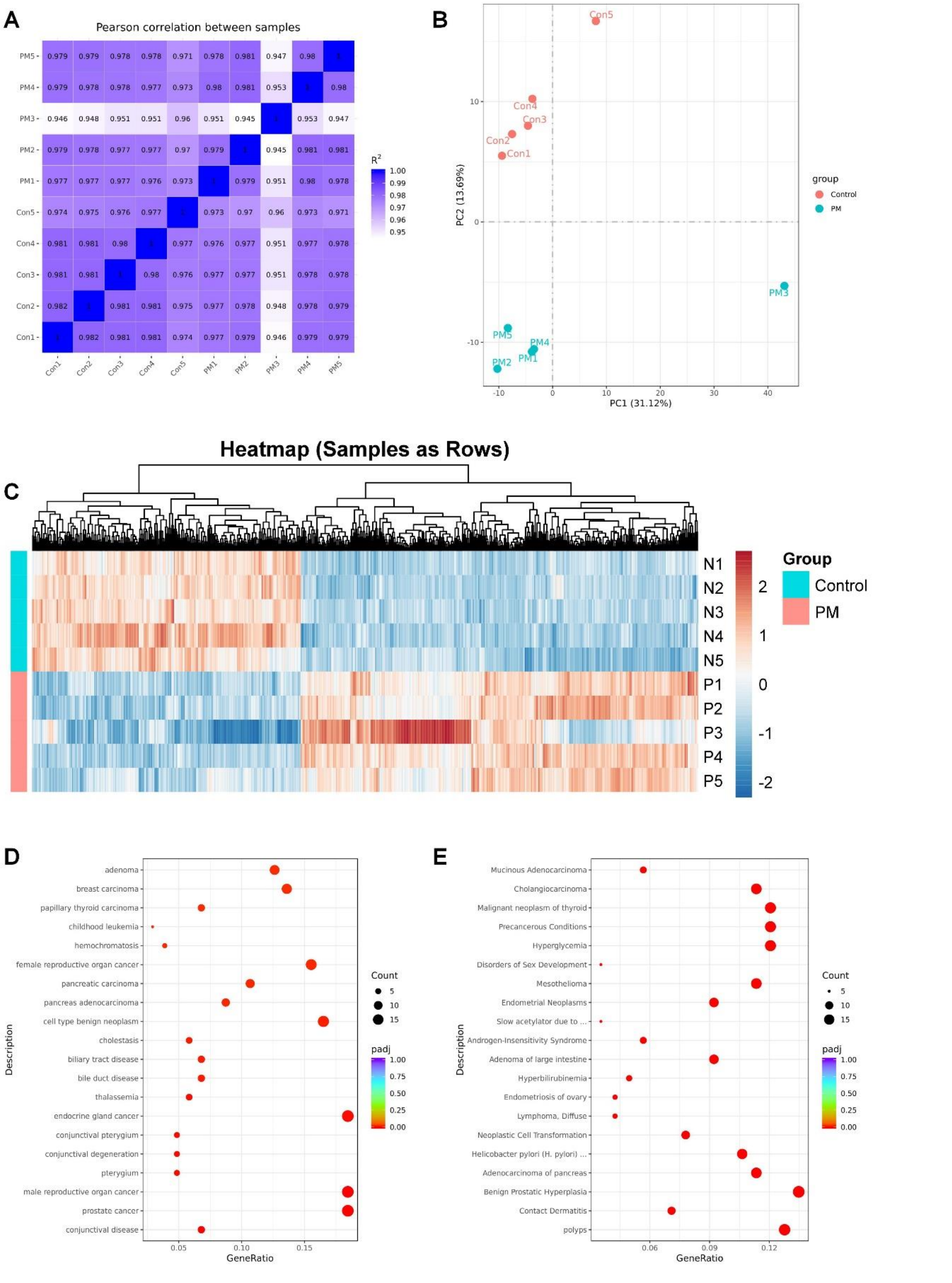

Figure S1 Transcriptomic Profiling Results: Sample sequencing data quality control. Correlation (A) and Principal Component Analysis (B) among all samples. (C) Heatmap of differentially expressed genes between $PM_{2.5}$ treatment and control groups. Log2FoldChange > 0, Adjust p-value <0.1. (D-E) bubble plot of top 20 terms enriched in the Disgenet analysis and DO analysis (E).
